# H3K4me3 demethylation by the histone demethylase KDM5C/JARID1C promotes DNA replication origin firing

**DOI:** 10.1093/nar/gkv090

**Published:** 2015-02-23

**Authors:** Beatrice Rondinelli, Hélène Schwerer, Elena Antonini, Marco Gaviraghi, Alessio Lupi, Michela Frenquelli, Davide Cittaro, Simona Segalla, Jean-Marc Lemaitre, Giovanni Tonon

**Affiliations:** 1 Functional Genomics of Cancer Unit, Division of Experimental Oncology, Istituto di Ricovero e Cura a Carattere Scientifico (IRCCS), San Raffaele Scientific Institute, Via Olgettina 60, 20132 Milan, Italy; 2 Molecular Medicine PhD Program, Vita-Salute San Raffaele University, Via Olgettina 58, 20132, Milan, Italy; 3 Laboratory of Stem Cell and Genome Plasticity in Development and Aging, Institute of Regenerative Medicine and Biotherapies, INSERM U1183, Montpellier University, Montpellier, France; 4 Centre for Translational Genomics and Bioinformatics, Istituto di Ricovero e Cura a Carattere Scientifico (IRCCS), San Raffaele Scientific Institute, 20132 Milan, Italy

## Abstract

DNA replication is a tightly regulated process that initiates from multiple replication origins and leads to the faithful transmission of the genetic material. For proper DNA replication, the chromatin surrounding origins needs to be remodeled. However, remarkably little is known on which epigenetic changes are required to allow the firing of replication origins. Here, we show that the histone demethylase KDM5C/JARID1C is required for proper DNA replication at early origins. JARID1C dictates the assembly of the pre-initiation complex, driving the binding to chromatin of the pre-initiation proteins CDC45 and PCNA, through the demethylation of the histone mark H3K4me3. Fork activation and histone H4 acetylation, additional early events involved in DNA replication, are not affected by JARID1C downregulation. All together, these data point to a prominent role for JARID1C in a specific phase of DNA replication in mammalian cells, through its demethylase activity on H3K4me3.

## INTRODUCTION

DNA replication is one of the most tightly regulated cellular processes, since even a modest disruption may have dire consequences for the cell. During each cell cycle, it must occur at the appropriate phase (1) and only once (2). Therefore, replication proceeds in temporally distinct and independent steps during the cell cycle (1). Already in late mitosis and early G1, a set of proteins, the origin recognition complex (ORC) proteins, bind to locations throughout the genome where DNA replication is going to ensue, the DNA replication origins (1). The subsequent recruitment of the minichromosome maintenance 2–7 hexameric complex (MCM 2–7) on these sites, at the onset of the S phase, leads to the formation of the pre-replication complex (pre-RC) (3,4). This event is known as origin licensing. The pre-RC is maintained inactive until the entry into S phase, when the transition from pre-RC to pre-initiation complex (pre-IC) is promoted by the activity of two different kinases. A serine-threonine kinase, CDK1 (cyclin-dependent kinase 1) phosphorylates the Sld3-related protein Treslin/ticcr (5). In parallel, DDK (Dbf4-dependent kinase) phosphorylates pre-RC components MCM 2–7 (6). These events promote the recruitment onto the DNA of the highly conserved proteins CDC45 and GINS (7), leading to the formation of the active CDC45/MCM2–7/GINS (CMG) holo-helicase complex (6,8–10). Active CMG promotes the elongation phase of DNA replication (10,11), proceeding bi-directionally to duplicate DNA.

During the elongation phase of DNA replication, the CMG helicase unwinds the DNA while the recruited DNA polymerases synthesize daughter strand DNAs (12). To improve polymerase processivity, the homo-trimer proliferating cell nuclear antigen (PCNA) localizes on DNA. PCNA is a ring-shaped complex that encircles the double helix forming a sliding clamp. PCNA firmly tethers polymerases to DNA, ultimately increasing their processivity from tens, to thousands of nucleotides (13,14).

In eukaryotes, DNA replication is initiated from a large set of origins (15). The order by which origins are fired is tightly regulated, with most origins firing early in S phase, fewer later and finally only a small subset in the final stages of the S phase (16). Unlike lower organisms, in metazoans the nucleotide sequence of DNA replication origins is not endowed with clearly defined features, and *cis-acting* genetic elements required for ORC binding and origin activity were not characterized until recently (15). Indeed, a consensus G-quadruplex-forming DNA motif that can predict potential positions of DNA replication origins in human cells genome-wide was identified (17), but determinants regulating positioning, usage efficiency and activation timing remains largely unknown. As such, in metazoan, the mechanisms regulating the coordinated firing of origins remain largely unknown. Recent evidence have suggested that the chromatin landscape surrounding replication origins may profoundly impact and regulate origin activity (18,19), suggesting that epigenetic changes on these DNA loci may be crucial for DNA replication. For example, nucleosomes modulate the accessibility of replication origin (20–22). Additionally, histone post-translational modifications including acetylation of histones H3 and H4 accelerate the timing of origin firing within the S phase (23,24). In yeast, histone H3 lysine 36 mono-methylation (H3K36me1), induced by the Set2 methyltransferase, is required for the recruitment of the Cdc45/GINS holo-helicase component Cdc45 (25). In eukaryotes, PR-Set7 adds one methyl group to H4K20, favoring the assembly of the core helicase (26,27).

The methylation of the lysine on position 4 of histone 3 (H3K4me1, 2 and 3) is one of the more versatile and widespread histone modifications. Histone post-translational modifications affecting this residue have been implicated, for the most part, in finely tuning gene expression on promoters (28). The potential role of H3K4 methylation on DNA replication remains controversial. In yeast, plants and mammalian cells, H3K4me2 and H3K4me3 are enriched on replication origins (29–31). Moreover, H3K4 di-methylation by the yeast Set1-containing COMPASS complex is required for proper S phase progression in yeast, while H3K4 tri-methylation is dispensable (29). Curiously, however, during DNA replication, a gradual loss of H3K4me3 has been reported in yeast on nucleosomes localized at early- and late-replicating regions (32). This reduction is an active process, since it relies on the active removal by the histone demethylase Jhd2/Kdm5 (32). Therefore, the functional role of H3K4me3 on replication origins remains largely unknown.

*JARID1C* belongs to the JARID subfamily of JmjC-containing proteins, together with RBP2 (*JARID1A*), PLU-1 (*JARID1B*) and SMCY (*JARID1D*) (33). The JmjC domain of *JARID1C* represents its catalytic moiety that specifically demethylates di- and trimethylated lysine 4 on histone 3 in a Fe(II) and α-ketoglutarate-dependent manner (34). The JmjN domain and the C5HC2 zinc finger are both important for assisting JARID1C catalytic activity (35). *JARID1C* contains also a BRIGHT domain and an AT-rich domain interacting domain (ARID) that binds DNA (36). Finally, one of the two PHD domains interacts with trimethylated lysine 9 on histone 3 (H3K9me3) (34).

*JARID1C* resides on the X chromosome. It is expressed in multiple human tissues, has a paralogue on the Y chromosome and is highly conserved across evolution (37). *JARID1C* is one of the few genes on the X chromosome escaping X-inactivation (37). *JARID1C* missense, frame-shift and nonsense mutations have been linked to X-linked mental retardation (XLMR) (38). Notably, most of the nucleotide substitutions identified so far cause partial or complete loss of function in the demethylase activity of the protein (34,39). JARID1C exerts a prominent role in neuronal development and function. Indeed, silencing of *JARID1C* expression in *zebrafish* causes brain-patterning defects as well as impaired dendrite development and significant neuronal cell death in rat neurons (34). JARID1C contributes to neuro-development through transcriptional repression. In fact, it was isolated as part of a transcriptionally repressive complex containing HDAC1/2, EHMT2 (G9a) and REST (40). This complex binds REST-responsive elements on neuronal specific promoters such as brain-derived neurotrophic factor (BDNF), SCG10 and SCN2A, inhibiting their transcription through JARID1C demethylase activity.

Recent evidence hints to a possible transcription-independent role for JARID1C. JARID1C was among 485 chromatin factors enriched at nascent versus mature post-replicative chromatin (41). Moreover, JARID1C interacts with PCNA through its PIP (PCNA-interaction protein) motif (42). These findings may suggest a potential role for JARID1C in DNA replication. We now provide functional evidence that JARID1C positively regulates origin function through histone 3 lysine 4 demethylation at DNA replication origins. As such, JARID1C demethylase activity promotes the timely formation of the pre-IC complex shepherding the binding of CDC45 and PCNA to chromatin. JARID1C-mediated H3K4me3 demethylation is a required step for proper origin functionality in metazoans. Based on our findings, we propose a new role for JARID1C in origin function and DNA replication.

## MATERIALS AND METHODS

### Cell culture

HeLa were cultured in Dulbecco's modified Eagle's medium (DMEM) (EuroClone) supplemented with 10% fetal bovine serum (EuroClone) and penicillin–streptomycin antibiotics (Sigma).

### shRNA mediated silencing of JARID1C expression

A pLKO.1 shRNA set targeting JARID1C was purchased from Open Biosystem (Thermo Scientific). The shRNAs numbers 3 (shRNA A) and 1 (shRNA B), the most efficient in silencing JARID1C expression, were used in this study. They have the following hairpin sequences and both recognize the coding sequence of human JARID1C:

### shRNA A

*CCGG***GTGACAGTAAACGGCACCT***TTCTCGAGAA***AGGTGCCGTTTACTGTCA***CTTTT*

### shRNA B

*CCGG***GCAGTGTAACACACGTCCA***TTCTCGAGAA***TGGACGTGTGTTACACTG***CTTTT*

Common structures of shRNA backbones are highlighted in italics. Non-targeting, scrambled shRNA (CTR shRNA) negative control was purchased from Sigma. Studies in HeLa cells except DNA combing were performed by transfection with Fugene HD™ (Promega) according to the manufacturer's instructions.

### Lentiviral production and transduction

To obtain lentiviral particles for the infection in the DNA combing experiments, HEK-293T cells were transfected with the CaCl_2_ method. To this end, a mix containing: 10 μg of transfer pLKO.1 vector, 6.5 μg of packaging vector Δr 8.74, 3.5 μg of Env VSV-G, 2.5 μg of REV, 300 μl of 0.1× TE, 150 μl of dH_2_O, 50 μl of 2.5M CaCl_2_ and 500 μl of 2× Hepes Buffered Saline (HBS) was added drop by drop on a 10 cm_2_ dish. After 18 h, the medium was changed and after additional 36 h the medium was collected, centrifuged to remove cell debris and ultracentrifuged. At this point the pellet of viral particles was resuspended in phosphate buffered saline (PBS) and stored at –80°C in small aliquots.

### Immunofluorescence

The GFP-JARID1C fusion protein was originated by inserting the GFP coding sequence upstream of JARID1C ORF in a pDEST51 expression vector (Invitrogen). Forty-eight hours after transfection with GFP-JARID1C fusion protein, HeLa cells were fixed in cold methanol. Cells were then permeabilized with 0.3% Triton X-100 (Sigma) in PBS for 10 min at room temperature (RT). After three PBS washes, aspecific binding sites were blocked by treatment with blocking solution (PBS/3% Bovine Serum Albumin (BSA)) for 30 min at RT. Staining with anti-PCNA primary antibody (sc-56, 1:100 dilution) was used to detect PCNA. Anti-mouse secondary antibody was prepared in PBS/3% BSA and was left 45 min at RT. Coverslips were mounted on glass slides with ProLong^®^ Gold Antifade Reagent with 4',6-diamidino-2-phenylindole, Invitrogen (DAPI). For confocal 3D analysis, a Leica Confocal – TCS SP2 Laser Scanning Confocal at the ALEMBIC (Advanced Light and Electron Microscopy Bio-Imaging Centre, San Raffaele Scientific Institute) facility was used. Stacks of 25–30 images were acquired for each field and analysed with ImageJ software.

### Analysis of bromodeoxyuridine/propidium iodide plots

A dual parameter histogram (BrdU/PI) of the cell cycle phase distribution in the different phases of the cell cycle was generated using FCS Express 4^®^ (De Novo Software). Calculations of the percentage of cells in G0/G1 and G2/M phases were obtained by gating on DNA content based on PI incorporation and by quantifying the number of cells with 2*n* (G0/G1) and 4*n* (G2/M) DNA content. S phase cells were quantified as positive for BrdU incorporation and with an intermediate 2*n*–4*n* DNA content. For quantitative representation in column diagrams the relative BrdU fluorescence was calculated by normalizing the BrdU signal of S-phase cells to the signal intensity of BrdU-negative G1 and G2/M cells. This was normalized on BrdU incorporation of control siRNA treated S-phase cells similarly calculated. For cell cycle progression of G1/S synchronized HeLa cells, a PI histogram plot was generated for each sample and compared with other time points through a 3D representation.

### Synchronisation of HeLa cells

Timings were set to obtain an optimal silencing of JARID1C at the time of release, using either double thymidine block or nocodozale treatment.

**Double thymidine block**. On average 80% of cells were in G1/S at the time of release. To follow the progression through the S phase, cells were pulse labeled with BrdU for 20 min before harvesting. Briefly, 1.5 × 10^5^ cells/six well were plated at low confluence (day −2). The same evening cells were washed and placed overnight in medium supplemented with 2 mM thymidine (Sigma) for 14–15 h. The following day (day −1), after extensive washes, cells were placed in serum-free medium. After 8–9 h (washout), a second 14–15 h 2 mM thymidine block was performed overnight. On day 0, at 1, 2, 3, 4, 5, 7 and 9 h after the release, cells were washed and detached with trypsin (EuroClone). Half of the cells were fixed for cell cycle analysis in 75% ethanol (Sigma) while the other half was pelleted for western blot analysis. Control asynchronous HeLa cells were collected at day 0 and processed as synchronized cells.

**Nocodazole block**. HeLa cells growing in log phase were treated for 14–15 h with 250 ng/μl nocodazole (Sigma). The following morning, after extensive washes, nocodazole-treated cell pellets were harvested (0 h) or replated in and harvested at 3 (G1), 9 (early S) or 12 (late S) h post-release.

### Caffeine and UCN-01 treatments

Caffeine (Sigma) was dissolved in water at 80°C at a concentration of 100 mM. At 72 h after transfection with shRNA plasmids, cells were treated with 5 mM caffeine diluted in medium for 2 h. At the end of the treatment, a 20-min pulse with 10 μM BrdU was performed. At this point, half of the cells were fixed in 75% ethanol for cell cycle analysis by BrdU/PI incorporation. Cells were incubated with UCN-01 (Sigma) at 500 nM for 2 h. A positive control for checkpoint activation was obtained by treatment of wt HeLa cells with 0.3 mM aphidicolin (Sigma) for 24 h.

### CSK fractionation

Triton X-100 extraction was performed as in (43). Briefly, release of chromatin-bound material was performed in multi-well plates by extraction with 0.5% T-CSK (10 mM PIPES pH 7, 100 mM NaCl, 300 mM sucrose, 3 mM MgCl_2_, 0.5% Triton X-100) for 5 min. Cells were then rinsed with CSK and PBS before fixation in 4% PFA. Protease and phosphatase inhibitors were included during extraction and following rinses (Complete, EDTA-free Protease Inhibitor Cocktail Tablets, Roche).

### SDS-PAGE and immunoblotting

Cells pellets were washed twice with ice-cold PBS, resuspended in laemmli sample buffer 1× + 100 mM Dithiothreitol (DTT) and sonicated with five 1 min on/off cycles with Bioruptor (Diagenode). Protein electrophoresis was performed on polyacrylamide gels (BioRad) and proteins transferred to nitrocellulose membranes (GE Healthcare, Amersham Hybond ECL) with a Mini Trans-Blot Electrophoretic Transfer System (Biorad). Lysis buffer was complemented with Protease Inhibitor Cocktail Tablets (Roche).

### Molecular DNA combing

Large-scale knockdown was performed through transduction of five T75 with cells at 70% of confluence with scrambled or J1C specific shA-coding lentiviral vector. Culture medium was changed 18 h after transduction and selection of infected cells was obtained with puromycin (Sigma), 2 μg/ml final concentration. After 72 h of selection, a small aliquot of cells was tested for JARID1C knockdown by western blot while the remaining cells were collected and treated for DNA combing. For specific measurement of replication fork velocity and inter-origin distances, cells were successively labeled for 15 min with 20 μM IdU (Sigma) and for 30 min with 100 μM CldU (Sigma) and collected for DNA combing, performed as previously described (44). Briefly, cells were collected after CldU labeling and embedded in agarose plugs (about 1.5 × 10^5^ cells/plug). DNA was stained with YOYO-1 (Invitrogen), and re-suspended in 50 mM MES (pH 5.7) after digestion of the plugs with agarase (Biolabs). DNA fibers were stretched on sylanized coverslips. Combed DNA fibers were denatured for 30 min with 0.5 N NaOH. IdU was detected with a mouse monoclonal antibody (BD44, Becton Dickinson; 1:20 dilution) and a secondary antibody coupled to Alexa 546 (A21123, Invitrogen, 1:50 dilution). CldU was detected with a rat monoclonal antibody (BU1/75, AbCys; 1:20 dilution) and a secondary antibody coupled to Alexa 488 (A11006, Invitrogen; 1:50 dilution). DNA was detected with an anti-ssDNA antibody (MAB3034, Euromedex; 1:100 dilution) and an anti-mouse IgG2a coupled to Alexa 647 (A21241, Invitrogen, 1:50 dilution). Immuno-detection of DNA fibers were analyzed on a Leica DM6000B microscope equipped with a CoolSNAP HQ CCD camera (Roper Scientifics). Data acquisition was performed with the ImageJ software. Box-and-whiskers graphs were plotted with Prism v5.0d (GraphPad Software). For all graphs, whiskers correspond to 10–90 percentile and the line near the middle of the box marks the median (50th percentile). Data not included between the whiskers are plotted as outliers (dots). Statistical analysis was performed in Prism v5.0d (GraphPad) using the Mann–Whitney non-parametrical test and the *χ*^2^ test for the replication figures.

### Chromatin immunoprecipitation

Chromatin was collected and purified as in (45). LB1, LB2 and LB3 were supplemented with Complete, EDTA-free Protease Inhibitor Cocktail Tablets (Roche). Lysates were sonicated with Bioruptor (Diagenode). Briefly, 300 μl aliquots of LB3 lysates were dispensed in Eppendorf 1.5 ml tubes and sonicated for 10 min (medium intensity, 30 s on, 30 s off). This sonication protocol allowed us to obtain products ranging from 300 to 500 bp. Upon spectrophotometric quantification of chromatin (Nanodrop), 50–100 μg of ready-to-use chromatin were used for each anti-human PCNA chromatin immunoprecipitation (ChIP), while 10–20 μg were used for each ChIP to histones. For each ChIP, 70 μl of Dynabeads protein G slurry (Invitrogen) were washed and incubated with 10 μg antibody (5 μg antibody for ChIP histones). Samples were purified with QIAquick PCR purification kit (Qiagen), following manufacturer's recommendations. DNA was eluted in 30–70 μl of TE buffer and 1–2 μl were used in qPCR Sybr^®^ Green qPCR kit (Invitrogen). One experiment (of the two performed) is reported in figures.

### ChIP qPCR analysis

To determine the enrichment obtained, we normalized ChIP-qPCR data for input chromatin (reported as % input in the figures).

**Table:** 

TOP1–5KB F	ATCTCCTTTCTTGAGCACCCAGCA
TOP1–5KB R	AACAGGATGTCCCACCTGACGTTT
TOP1 ori F	TTATGCAAATCACAGCGGAG
TOP1 ori R	CAGACCCCAGCAGTTGTGTA
TOP1 +10 kb F	AGGAATGTGCCCACAATAGGGTTG
TOP1 +10 kb R	AAGACCTAGCCATTGAGCAGAAGC
MCM4 −5 kb F	GTGCATCTGTCCACAGCAAAGGTT
MCM4 −5 kb R	AAGCTGGGTGGATGTGAATCCTGT
MCM4 ori F	AAGGGATAAAGAAGCACACGCTCC
MCM4 ori R	AAACCAGAAGTAGGCCTCGCTCG
MCM4 +5 kb F	AGCACGTGCATGATTCTGTAGGGT
MCM4 +5 kb R	GAGTCAGGGTAACGGTCAAAGAAG
LaminB2 −5 kb F	CCTTGGGTGCGTGTTTCTTGCTAA
LaminB2 −5 kb R	AACACCAGCTACCTCCACACAGAA
LaminB2 ori F	AGTGACCTTTTTCCTTGGGG
LaminB2 ori R	GCTGGCATGGACTTTCATTT
LaminB2 +5 kb F	GGATGTACTTGGGCGTGAACTTGT
LaminB2 +5 kb R	CCGTGTCTTCCAGGATCAGTCTCT
β-Globin −15 kb F	TGTCCCATCCAGGTGATGTTCTCA
β-Globin −15 kb R	GTAGTCAGTGAGTCTAGGCAAGATG
β-Globin ori F	AATCTATTCTGCTGAGAGATCACAC
β-Globin ori R	CCACTTGCAGAACTCCCGTGTAC
β-Globin +15 kb F	ACCCTTCACACATGAGAGTCACCA
β-Globin +15 kb R	TCAAACCCAAGAGGAGCAGTGAGT
c-MYC F	TATCTACACTAACATCCCACGCTCTG
c-MYC R	CATCCTTGTCCTGTGAGTATAAATCATCG
PTGS2 F	GTTCTAGGCTGGTGTCCCATTG
PTGS2 R	ACTCCCAGCGGAGACAATTT
NETO1 F	GCTGAGTGTGGCCTTAAGAGGA
NETO1 R	AAGTTGCATGAATGCTTCTTTTT
SLITRK6 F	GGAGAACATGCCTCCACAGTCT
SLITRK6 R	CACTGGTGGAGTAGGGCAAA

All primers were from (27), except c-MYC (46) and PTGS2, NETO1 and SLITRK6 (47). All primers were used at a final concentration of 400 nM.

### Analysis of H3K4me distribution by Repli-Seq profiles

We downloaded raw sequence reads for H3K4me1, H3K4me2, H3K4me3 and Input DNA from Short Read Archive (submission ID SRA037396), both for HeLa-S3 and K562 cell lines (48). We aligned reads to human genome (version hg19) using bwa aligner (49). We downloaded Repli-Seq smoothed profiles for HeLa-S3 and K562, these are represented in ENCODE in intervals 1 kb long, each interval is assigned a value between 0 and 100 indicating the fraction of S-phase at which DNA is replicated. We calculated the read coverage of histone marks and input DNA over every interval, matching data by cell line, and normalized the counts by total library sizes. In order to summarize coverages by Repli-Seq timing, we binned Repli-Seq profile values in 100 equal intervals and summed normalized coverages for regions found in each timing bin. We reversed the summary matrix by timing, as higher Repli-Seq values correspond to earlier events. Data for each histone marker were plotted in order to depict the distribution of DNA under a specific feature over replication time. Distribution of Input DNA was plotted as well.

In order to evaluate statistical significance in the difference of histone mark and DNA curves, we applied one-way Kolmogorov–Smirnov (KS) test on two samples. We extracted the D-statistic by calculating the maximum of the difference between the cumulative distributions, calculating the KS-test *P*-values assuming sample size being equal to 100.

### Generation of JARID1C mutant and rescue experiments

JARID1C cDNA (pCMV-SPORT6 expression vector, Open Biosystems) was cloned in a MSCV vector backbone. MSCV-wtJARID1C vector was used as template plasmid to engineer the H514A/A388P mutant (referred as ‘mut’) by two different rounds of site-directed mutagenesis, according to the instructions included in the Stratagene mutagenesis kit. Briefly, for each round, 5, 10, 20 and 50 ng of template plasmid were used for PCR with mutated primers (sequences are listed below). After the site-directed mutagenesis reaction, the parental, methylated plasmid was digested with DpnI restriction enzyme. Five to ten microliters of PCR-mutated plasmids were used to transform 100 μl of Mach1^®^ competent cells (Invitrogen). Plasmid DNA was extracted and verified by sequencing. For the second round of site-directed mutagenesis, A388P mutated primers (listed below) were used with the MSCV-JARID1C H514A single mutant backbone as a template. Rescue experiments were performed by transient transfection with shRNA plasmids. Briefly, pLKO.1 containing scrambled and JARID1C specific (shRNA A) shRNA hairpins were transfected as reported above with jetPRIME™. Twenty-four hours later, 300 ng of MSCV-LacZ, MSCV-wtJARID1C or MSCV-JARID1CdoubleH514A/A388P were transfected for each six-well plate. Together with rescue plasmids, an unmatched GFP-encoding plasmid was transfected to obtain a 1:3 jetPRIME™ DNA ratio. After 48 h from rescue and 72 h from pLKO.1 transfection, cells were detached, pelleted for lysates, cell cycle analysis or ChIP.

**Table:** 

	**Forward**
**H514A**	GGTCTTCTCAGCCTTTTGCTGGGCTATTGAGGATCACTGGAG
	**Reverse**
	CTCCAGTGATCCTCAATAGCCCAGCAAAAGGCTGAGAAGACC
**A388P**	**Forward**
	GCCTTTGGCTTTGAGCAGCCTACCCGGGAATACACTCTG
	**Reverse**
	CAGAGTGTATTCCCGGGTAGGCTGCTCAAAGCCAAAGGC

### Antibodies

For cytofluorimetric analysis of BrdU incorporation, directly conjugated Alexa Fluor-647 antibody from BD Pharmingen was used. Immunofluorescent analysis of S-phase cells was performed with mouse anti-BrdU antibody from Becton Dickinson (dilution 1:100). Anti-FLAG antibody was from Sigma (M2 clone, anti-mouse, 1:1000). As secondary antibody, anti-mouse FITC from Molecular Probes was used (Alexa Fluor-488, Cat. # A21202, 1:1000). For immunoblotting of nitrocellulose membrane, primary antibodies used were: α-huJARID1C (Abcam Cat. # 72152, dilution 1:2000); α-PARP1 (Abcam, Cat.# 32071, dilution 1:1000); α-CHK1Ser345 (Cell Signaling, Cat. # 2341, 1:1000 dilution); α-total CHK1 (Cell Signaling, Cat. # 2345, 1:1000); α-MEK2 (Cell Signaling, Cat. # 9125, 1:1000); α-LAMIN B (Sc-6216, Clone C20, 1:500); α-MCM5 (Abcam, Cat. # ab17967, 1:2000); α-MCM2 (Abcam, Cat. # ab4461, 1:1000); α-CDC45 (Santa Cruz, Cat. # C20, 1:500); α-PCNA (Santa Cruz, Cat. # sc-56, 1:500); α-cyclin A (Santa Cruz, Cat. # sc-751; 1:1000). Loading controls used were horseradish peroxidase (HRP)-conjugated α-β-ACTIN (BD Biosciences, 1:50 000) and anti-Histone H3 (Abcam, 1:25 000). Secondary antibodies used were HRP-conjugated α-mouse and α-rabbit (both from GE Healthcare). After immunoblotting, signals were detected using ECL Detection System (Amersham).

### ChIPs

Antibodies used were as follows: anti-H3K4me3 (Millipore, 5 μg/IP), anti-PCNA (Santa Cruz, Cat.# sc-56, 5 μg/ChIP), anti-H3K4me1 (Millipore, 5 μg/IP). Control isotypic IgGs were from Santa Cruz Biotechnology.

### Quantification of proteins in western blot experiments

Proteins were quantified using the densitometry function of the ImageJ software (http://imagej.nih.gov/ij/index.html), normalized to H3 levels within the same sample and expressed as arbitrary units.

### Statistical analysis

Averaged data were compared with the use of unpaired Student's *t*-test. To compare CTRsh and JARID1CshA distribution of replication figures, Chi-square was calculated. A *P* < 0.05 was considered as statistically significant. (NS, not significant; *P < 0.05; **P < 0.01; ***P < 0.001). Statistical analyses were performed with GraphPad Prism 4 software (GraphPad Software, San Diego, CA) and with R.

## RESULTS

### Depletion of JARID1C causes a checkpoint-independent S-phase delay

**Figure 1.**
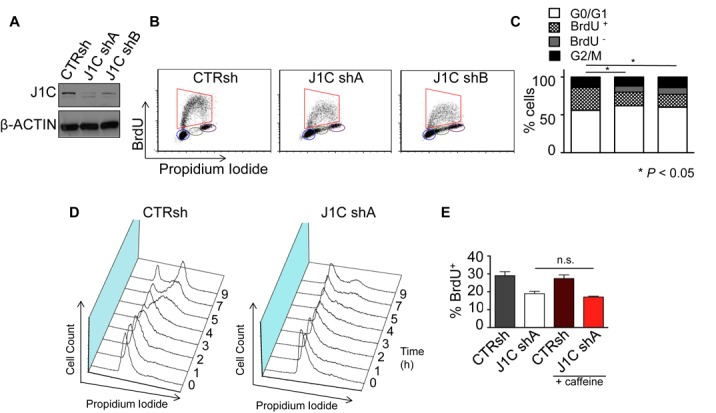
JARID1C depletion causes a checkpoint-independent S-phase delay. HeLa cells knocked-down with a scrambled shRNA (CTRsh) or two different shRNAs against *JARID1C* (J1C shA and J1C shB) were evaluated for (**A**) JARID1C and β-ACTIN expression; (**B** and **C**) BrdU incorporation. The percentage of cells in each phase of the cell cycle was determined by dual color PI/BrdU fluorescence-activated cell sorting (FACS) as stated in ‘Materials and Methods’. section *P < 0.05 between the two conditions using *X*^2^ test over distribution. (**D**) Control (CTRsh) and silenced (J1CshA) HeLa cells were G1/S synchronized with thymidine, released and monitored at 0–9 h post-release for cell cycle progression by PI staining. Histograms represent cell count versus DNA content as quantified by PI incorporation (see ‘Materials and Methods’ section). Numbers depict hours after the release. 3D representations show one representative experiment of two performed. (**E**) BrdU incorporation of CTRsh and J1CshA cells mock treated or treated with 5 mM caffeine for 2 h before BrdU pulse and harvesting. Bars represent mean ± SEM of three independent experiments. *n.s.*, non significant.

To determine the potential role of JARID1C in S-phase progression and DNA replication, we first explored whether JARID1C is involved in cell cycle progression. To this end, its expression was down-regulated in HeLa cells with two different shRNAs (Figure 1A) and cell cycle profiles of control and silenced cells after PI/BrdU incorporation were analyzed. Cells silenced for *JARID1C* failed to progress into S phase, as shown by a reduction in BrdU incorporation and by the accumulation of BrdU^−^ cells in S phase (Figure 1B). In addition, JARID1C depletion reduced overall DNA synthesis, since the fluorescence intensity of JARID1C-depleted BrdU incorporating cells was reduced as compared to control cells (1.48X and 1.64X fold decrease of BrdU fluorescence) (Supplementary Figure S1A). Interestingly, increased JARID1C expression had the opposite effect, promoting BrdU incorporation (Supplementary Figure S1B). Prolonged JARID1C depletion ultimately leads to apoptosis, as shown by increased cleavage of PARP1 (Supplementary Figure S1C). To further demonstrate a role for JARID1C in S-phase progression, we synchronized HeLa cells at the G1/S border by a double thymidine block (DTB). DNA content and BrdU incorporation analysis of released cells indicated that the entry into S phase of *JARID1C* knocked-down cells was impaired (Figure 1D and Supplementary Figure S1D). In addition, S-phase progression was defective. In fact, while control cells progressed to G2/M stage after 9 h, the majority of *JARID1C*-depleted cells failed to complete S phase and reach G2/M (Figure 1D). In all, these results suggest that cells devoid of JARID1C fail to efficiently enter and progress through the S phase. This defect results in S-phase lengthening, impaired BrdU incorporation and eventually cell death, suggesting a potential role for JARID1C in DNA replication.

A delay in the progression through the S phase may be elicited by the activation of DNA damage, followed by the activation of the S-phase checkpoint, mediated by the phosphatidyl inositol 3′ kinase-related kinases (PIKKs) ATM and ATR (50). This checkpoint provides a transient cell cycle arrest during the S phase allowing cells to repair the damage, complete DNA replication and proceed to mitosis (51,52). To exclude the involvement of this checkpoint in hampering S-phase progression, we exposed cells to caffeine, an established inhibitor of ATM and ATR as well as UCN-01, a CHK1 inhibitor (53,54). Both treatments failed to restore normal progression through S phase in JARID1C silenced cells (Figure 1E and Supplementary Figure S1E). Indeed, no CHK1 activation was observed upon JARID1C depletion (Supplementary Figure S1F). Taken together, these results imply that knockdown of *JARID1C* causes a checkpoint-independent blockade of S-phase progression that ultimately induces apoptosis.

### JARID1C is cell cycle regulated and localizes at sites of DNA synthesis

**Figure 2.**
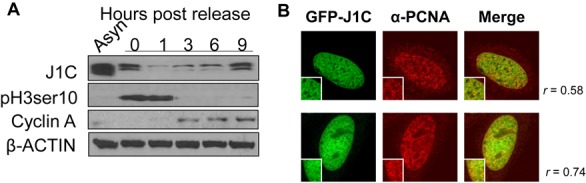
JARID1C is cell cycle regulated and co-localizes with PCNA. (**A**) Western blot analysis of JARID1C expression in nocodazole synchronized HeLa cells at different time points after the release. Phosphorylated Ser10 on histone H3 (pH3ser10) and cyclin A denote mitotic and S phase cells respectively. β-ACTIN is the loading control. (**B**) Confocal immunofluorescence of GFP-JARID1C and PCNA in early S phase HeLa cells (63×). Merged images show in yellow the co-localization of GFP-JARID1C with PCNA foci corresponding to early DNA replication sites. Magnifications show representative co-localization sites. Pearson colocalization coefficients are reported for each merged image.

Our data suggest a prominent involvement of JARID1C during the cell cycle, and specifically on the S phase. We thus asked whether JARID1C expression is cell cycle regulated. To this end, we synchronized cells with nocodazole and studied JARID1C expression at different time points after the release from the mitotic block. Remarkably, JARID1C expression was low in G1 cells and gradually increased when cells proceeded through S phase, with a peak of expression at 9 h post-release (Figure 2A). Interestingly, this time point corresponds to the early stages of S phase, as determined by the loss of phosphorylated Serine 10 of H3 and the increased expression of cyclin A (Figure 2A and Supplementary Figure S3A). In addition, we evaluated JARID1C binding to chromatin during the cell cycle. After G1/S synchronization, we observed a bimodal binding of JARID1C to chromatin as cells progress through S phase into mitosis. Specifically, JARID1C binding increased as early S proceeded (1 and 2 h timepoints, Supplementary Figure S2A). Interestingly, this increase was accompanied by overall reduction of H3K4me3 levels on chromatin. This is consistent with an active role of the H3K4me3 demethylase activity of JARID1C during S-phase progression (Supplementary Figure S2B). In addition, JARID1C bound chromatin also during the G2/M phase, suggesting an additional, origin-independent role for JARID1C during the cell cycle (4 h timepoint, Supplementary Figure S2A).

We next determined whether JARID1C localizes to sites of DNA synthesis in mammalian cells. PCNA is the fundamental clamp of the DNA replication machinery (13) and as such shows the characteristic patterns of DNA replication sites during S phase (55). On these premises, we assessed whether JARID1C co-localized with PCNA. Confocal immunofluorescence analysis demonstrated a significant degree of co-localization of *GFP-JARID1C* with early, PCNA positive DNA replication sites (Figure 2B and Supplementary Figure S2C). These data suggest a direct involvement of JARID1C at early sites of DNA replication.

### JARID1C participates to replication initiation

**Figure 3.**
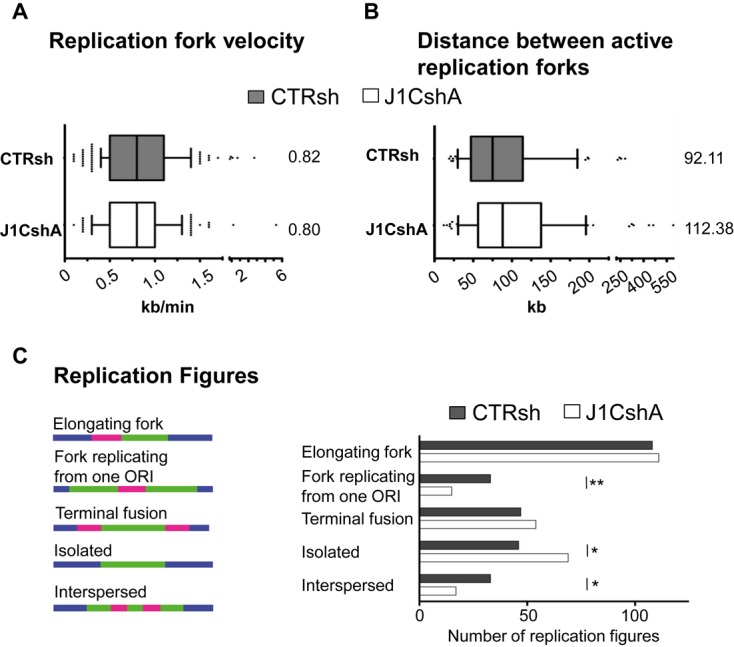
JARID1C depletion impacts on replication initiation. Seventy-two hours after transduction CTRsh or J1CshA cells were pulse-labelled with IdU (15 min) followed by CldU (30 min) and analysed by DNA combing for (**A**) measurement of replication fork velocity (*n* = 200) and (**B**) IOD (*n* = 100) (see ‘Materials and Methods’) section. Median values are indicated on the right. Statistical analysis was performed using unpaired Student's *t* test. (**C**) Pulse-labeled CTRsh and J1CshA treated cells were analysed by molecular DNA combing. Five classes of replication structures were defined. Left, representative classes of replication structures are shown and right, relative occurrence events for the different classes were scored. The number of replication structures analysed are *n* = 200 both for CTRsh than for J1CshA. *P < 0.5, **P < 0.01 between the two conditions using *X*^2^ test over distribution.

To further substantiate the direct role of JARID1C in DNA replication, we explored whether *JARID1C* silencing caused a direct hindrance in the execution of the DNA replication program. To this end, we performed *in vivo* DNA fiber labeling and molecular DNA combing analysis (56). Delayed DNA replication could stem from a lower frequency of initiation or a slower rate of fork progression. DNA combing revealed that fork velocity was not significantly reduced in *JARID1C*-knocked-down cells, when compared with control cells (Figure 3A). Indeed, also the lengths of replicated DNA tracks were not significantly different between control and silenced cells (Supplementary Figure S3B). These data indicate that *JARID1C* depletion does not impact fork progression and thus DNA replication elongation. We then analysed the distribution of active origins. Inter-origin distance (IOD) was on average increased in cells knocked-down for *JARID1C* (Figure 3B). Moreover, analysing the distribution of replication figures, we detected a significant reduction in the proportion of forks replicating from one ORI (*P* < 0.01), associated with an increase in the proportion of isolated forks (*P* < 0.05) and with a reduced number and increased skewedness of interspersed active origins (*P* < 0.05), with the appearance of IODs exceeding 300 kb (Figure 3C and Supplementary Figure S3C). Altogether, these results suggest that upon JARID1C downregulation a consistent subgroup of origins failed to be activated (57). As a consequence, significant portions of the genome might be replicated later than usual or stay un-replicated in *JARID1C*-depleted cells. Indeed, silencing of *JARID1C* phenocopies yeast strains lacking components of the initiation complex such as ORCs and Sic1 (58,59). All together, these evidence point to a direct role of JARID1C in the early events of DNA replication, namely pre-RC to pre-IC transition and origin firing, with no apparent effect on fork progression.

### JARID1C depletion causes aberrant H3K4 methylation at active origins of replication

**Figure 4.**
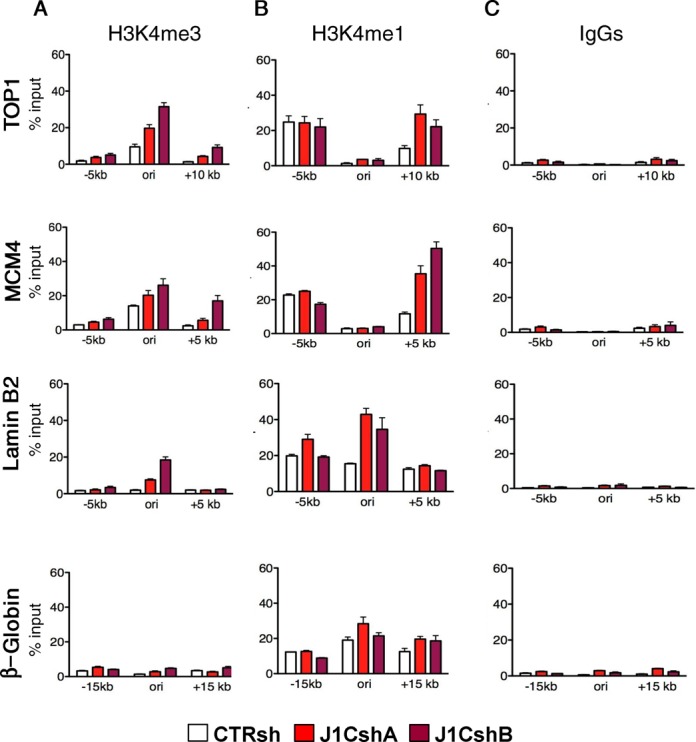
JARID1C depletion causes aberrant H3K4methylation at active DNA replication origins. Chromatin of control and silenced HeLa cells was immunoprecipitated with (**A**) anti-H3K4me3, (**B**) H3K4me1 specific antibodies or (**C**) isotipic IgGs used as controls. qPCR analysis on immunoprecipitated chromatin was performed with primers against the DNA replication origins *TOP1, MCM4, Lamin B2* and *β-globin*. Results are expressed as percentage of input (% input). The error bars represent SEM of a representative experiment of two performed.

JARID1C removes methyl groups from H3K4me2 and H3K4me3 (34). Since JARID1C downregulation impacts DNA replication and more specifically origin firing, we reasoned that the effect of JARID1C on DNA replication and origin firing might be mediated by its demethylase activity. Accordingly, we first explored whether JARID1C loss impacted on H3K4me3 methylation levels on well-characterized replication origins. After nocodazole synchronisation and release, we enriched cells in early S phase. With ChIP experiments, we then asked whether there was any enrichment for H3K4me3 within or nearby the extensively characterised, early-replicating origins, *Top1, MCM4* and *Lamin B2*, active in early S phase, and one late-replicating origin β*-*globin, used as control (23,24,60–62). We found that H3K4me3 was enriched on active DNA replication origins, when compared with flanking regions (Figure 4A and C). Moreover, silencing of *JARID1C* with two different shRNAs increased H3K4me3 levels at all the three active early-replicating origins tested. H3K4me3 levels on flanking regions were only modestly affected by JARID1C knockdown. In the late-replicating, inactive β*-*globin origin, H3K4me3 presented low levels and did not increase notably after *JARID1C* knock-down. As controls, we assayed also H3K4me1, a histone mark not targeted by JARID1C (34) (Figure 4B and C). Overall, in the active origins, no enrichment for H3K4me1 was evident, when compared with flanking regions, nor consistent increases in its levels upon *JARID1C* knock-down. Surprisingly, hence, these data suggest that the untimely and inappropriate increase of H3K4me3 on actively replicating origins may impact on proper DNA replication. As such, JARID1C may be required at each cell cycle to finely tune H3K4me3 levels on active replicative origins, thus regulating fork activation and firing.

We then asked whether JARID1C might exert a similar role in the activation of late-replicating DNA replication origins. We thus synchronised HeLa cells in late S phase and evaluated H3K4me3 levels at the β*-*globin DNA replication origin (Supplementary Figure S4A and B). In this specific cell cycle phase, as expected, JARID1C reduction did not impact on the levels of H3K4me3 on early-replicating *Top1* and *MCM4* origins. Interestingly, in addition, H3K4me3 was not significantly detected at active late-replicating β*-*globin origin, nor was JARID1C silencing affecting overall H3K4me3 levels during their activation (Supplementary Figure S4A and B). A similar pattern was present at three additional active late-replicating regions, namely *PTGS2, NETO1* and *SLITRK6* origins (Supplementary Figure S4C) (47). To corroborate these data, we explored the Repli-seq data in Encode. Remarkably, we found a significant enrichment for H3K4 methylation marks at early replicating origins in HeLa cells, while in late S, H3K4me2 and me3 were depleted at replicating origins (Supplementary Figure S5). We extended this analysis to the ENCODE data on the leukaemia cell line K562. Consistently to the data in HeLa cells, early origins were specifically enriched for methylated H3K4 histone marks. In all, these data suggest that in mammalian cells methylation of H3K4 is present for the most part at early-replicating regions. At these genomic regions, we propose that JARID1C is required to finely modulate H3K4me levels, thus driving their proper activation.

### JARID1C contributes to proper CMG formation and efficient PCNA loading on chromatin through H3K4me3 demethylation

**Figure 5.**
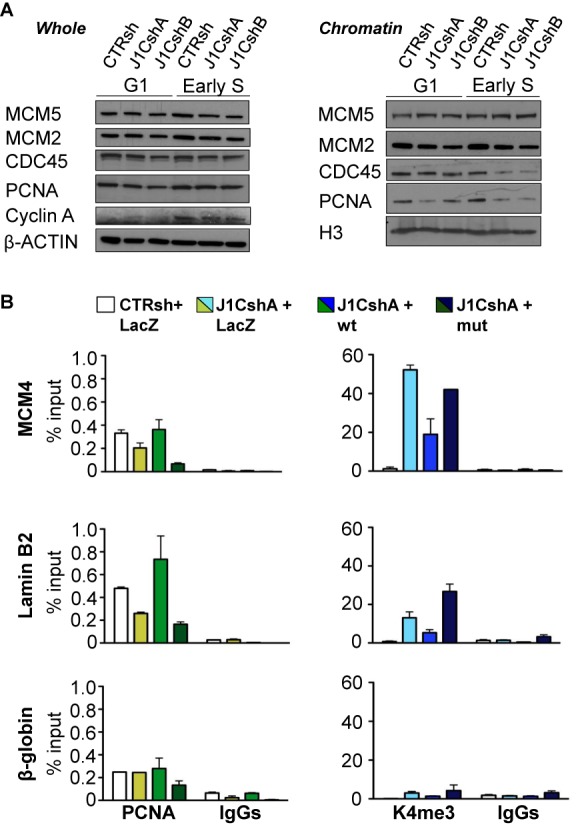
JARID1C contributes to proper CMG formation and efficient PCNA loading on chromatin through H3K4me3 demethylation. (**A**) left, CTRsh and J1CshA/shB silenced cells were G1 and S synchronized by nocodazole and released after 3 and 9 h, corresponding to enrichment of cells in G1 and S, respectively. Total cell lysates were analyzed for expression of the replication proteins MCM5, MCM2, CDC45 and PCNA by Western blot. Expression of cyclin A was used to evaluate progression from G1 to S phase. Expression of β-ACTIN was used as a loading control; right, chromatin enriched fractions of cell lysates from CTRsh, J1CshA and J1CshB cells were analyzed by western blot. (**B**) Chromatin of rescued control and down-regulated cells was immunoprecipitated with anti-PCNA and anti-H3K4me3 antibodies. Isotipic IgGs were used as controls. qPCR analysis on immunoprecipitated chromatin was performed with primers against MCM4, Lamin B2 and β-globin DNA replication origins, respectively. Results are expressed as percentage of input (% input). The error bars represent SEM of a representative experiment of two performed.

Based on our data, JARID1C depleted cells undergo impaired origin activation. This phenotype may be due to defective licensing, as reported for knockdown of ORC, Cdc6, Ctd1 or MCM2–7 by RNAi (63–65). Alternatively, impaired initiation may result from defective firing, as described for Sic1, ORC1, 2 and 5 initiation mutants in yeast (58,59). To dissect at which step of DNA replication initiation JARID1C acts, we studied the binding of factors involved in origin licensing and activation (1). To this end, cells were synchronised with nocodazole and then released, in order to enrich the population in early S-phase cells. We initially studied the binding of licensing factors in control and JARID1C depleted cells, assaying the chromatin binding of the most representative MCM2–7 components, MCM2 and MCM5, by western blot analysis of chromatin-bound fraction (43). JARID1C down-regulation did not affect the total levels of MCM2 and MCM5 present in the cells, nor, importantly, there was any significant difference in the association of MCM2 and MCM5 to chromatin between control and *JARID1C* silenced cells (Figure 5A). These results suggest that JARID1C is not involved in fork licensing. We thus explored origin firing. The loading of the CDC45 and GINS components onto the pre-RC (8–10) is the key event orchestrating origin firing (10,11). Indeed, reduced binding to early S-phase chromatin of either of these two components causes impaired initiation (66,67). Interestingly, *JARID1C* knockdown reduced the loading of CDC45 on chromatin of S -phase cells (Figure 5A, right and Supplementary Figure S6A), despite no apparent direct interaction between the two proteins (data not shown). Importantly, CDC45 protein levels in whole-cell extracts were not affected by *JARID1C* down-regulation (Figure 5A, left). These results support a role for JARID1C in the events that follow pre-RC formation, namely pre-IC activation through CDC45 binding.

The recruitment of CDC45 to the pre-RC complex is mediated by the serine/threonine kinase CDK1 (7). To exclude that JARID1C may affect CDC45 loading on DNA through CDK1, we assayed the total protein levels, as well as the phosphorylation of CDK1 at tyrosine 15 (Y15) (1). We did not detect any change in total CDK1 nor in the levels of its inhibitory Tyr15 phosphorylation as a result of JARID1C knockdown, suggesting that JARID1C regulates CDC45 binding to chromatin independently from CDK1 activity (Supplementary Figure S6B).

H4 acetylation contributes to the final step of pre-RC assembly (23,62) and to the activation of origins in early S phase (24,60). We did not detect any noteworthy difference in H4 acetylation in origins of control and *JARID1C* silenced cells, with higher levels on active early *MCM4* and *Lamin B2* origins (Supplementary Figure S6C). These data suggest that H4 acetylation may ensue independently from the activity of JARID1C, and does not depend upon JARID1C-mediated H3K4me3 demethylation during origin activation.

During the elongation phase of DNA replication, processivity of polymerases in DNA synthesis is promoted by PCNA. PCNA stably associates to the elongating replication fork and is bound to chromatin after CMG formation (13,14). Moreover, JARID1C directly interacts with PCNA ((42) and data not shown). Based on these data, we explored whether the knockdown of *JARID1C* altered PCNA binding to chromatin. Despite similar PCNA levels between down-regulated and control cells (Figure 5A, left), considerably less PCNA was associated with chromatin in *JARID1C* depleted cells (Figure 5A, right and Supplementary Figure S6A). To exclude that the decrease in CDC45 and PCNA was not due to a failure of progression into S phase, we assayed for E2F-induced cyclin A levels, a marker of S phase. Importantly, cyclin A was similarly expressed in control and silenced cells (Figure 5A, left). All together, these data suggest that *JARID1C* knockdown impacts on fork firing and on efficient DNA synthesis. *JARID1C* depletion prevents the loading to S-phase chromatin of proteins that are crucial for origin activation and replication elongation, such as, respectively, CDC45 and PCNA.

### PCNA binding to DNA is mediated by JARID1C-induced H3K4 demethylation

To conclusively demonstrate that the demethylase activity of JARID1C is required for its activity on DNA replication, we engineered a *JARID1C* point mutant lacking enzymatic activity (‘mut’, see ‘Materials and Methods’ section). We then performed ChIP experiments in nocodazole-released early S-phase CTRsh and J1CshA cells after rescue with vectors encoding for LacZ, wild-type JARID1C (‘wt’) or the ‘mut’ JARID1C. Of note, wt and mutant JARID1C were expressed at similar levels (Supplementary Figure S7A). Wt but not mutant JARID1C complemented BrdU incorporation in silenced cells to levels similar to control cells (Supplementary Figure S7B). We then evaluated whether CDC45 and PCNA binding to active origins was dependent on JARID1C demethylase activity. Unfortunately, the antibody used for CDC45 specific ChIP did not yield a significant enrichment over IgG. As anticipated, PCNA binding was reduced following *JARID1C* knockdown in three over four active early-replicating origins tested (*MCM4, Lamin B2* and *c-MYC* origins) (Figure 5B and Supplementary Figure S8A). H3K4me3 instead robustly increased upon JARID1C silencing (Figure 5B and Supplementary Figure S8A). Surprisingly, JARID1C knockdown increased PCNA binding to the early-replicating origin TOP1, while enhancing, at the same time, H3K4me3 levels. Both the reduction in PCNA and the increase in H3K4me3 were not apparent on the β*-*globin inactive late-origin. The reintroduction of wt JARID1C in cells downregulated for the gene rescued the phenotype, with an increase in PCNA binding and in parallel a reduction in H3K4me3 enrichment at the *MCM4, Lamin B2* and *c-MYC* origins, with no change in PCNA total protein levels (Figure 5B and Supplementary Figure S8B). Again, no differences were present in the β*-*globin DNA replication origin for both PCNA binding and H3K4me3 levels. The mutant itself was unable to rescue the binding of PCNA to *MCM4, Lamin B2* and *c-MYC* origins while, as expected, failed to reduce H3K4me3 levels. Notably, in some instances, mutant JARID1C appeared to cooperate with JARID1C downregulation in reducing PCNA binding, and increasing H3K4me3 levels, suggesting a potential dominant negative activity of this mutant toward the JARID1C remaining after knockdown. These data suggest that JARID1C drives efficient PCNA loading on chromatin at active replication origins through efficient H3K4me3 demethylation.

## DISCUSSION

We report here that the histone demethylase JARID1C prominently regulates DNA replication, finely modulating H3K4me3 levels at active, early replicative origins. We also provide evidence that JARID1C is involved in a specific step of DNA replication, namely early origin firing, dictating the binding of crucial players implicated in origin firing and DNA polymerase processing such as CDC45 and PCNA.

Chromatin remodelers are best characterised for their role in the regulation of gene expression (28). For example, Polycomb (PcG) and Trithorax-group (TrxG) proteins, respectively hinder or allow access of the transcriptional machinery toward gene promoters (68). The multilevel regulation of transcription involves not only promoters, but also other genomic elements such as enhancers (69), introns or exons (70), with the ultimate goal of shaping transcription of multiple genomic loci and indirectly coordinating developmental, metabolic and cell signalling programs (71). More recently, additional, transcription-independent roles have emerged for chromatin remodelers, including signaling at DNA damage sites and transmission of the parental nucleosomal features to daughter cells. Recent data support the notion that a crucial cellular process such as DNA replication is also directly governed and regulated by epigenetic mechanisms, independently from expression regulation (72). In fact, mechanisms regulating origin licensing and firing in metazoans remain largely unknown (15). The chromatin landscape surrounding replication origins is emerging as significantly influencing origin activity (18,19). For instance, specific post-translational modifications of histones H3 and H4 were shown to impact on origin licensing and firing (23–27). For example, acetylation levels of histone H4 at replication origins contribute to the successful and timely activation of origins (16,62). Importantly, acetylation at H4 undergoes coordinated regulation by the HDAC RPD3 and the HAT HBO1. Together, these enzymatic activities on H4 positively regulate both the licensing and the firing of DNA replication origins (16,62). In regard of lysine 4 on histone 3, evidences indicate that di and tri-methylation by specific HMTs in yeast replication origins contribute to S-phase progression and faithful duplication of the genome (29). Concomitantly, it was shown that H3K4me3 is removed by Jhd2/Kdm5 during S phase (32), thus supporting the hypothesis that lysine 4 on histone 3 undergoes a concerted regulation of its methylation status during DNA replication. Previous studies associated abnormal histone methylation at other lysine residues such as lysine 20 on histone H4 (H4K20) and lysine 36 on histone H3 (H3K36) with aberrant S-phase progression and S-phase lengthening in mammals (25,26,73). Here, we provide evidence that loss of JARID1C exerts an essential role in mammalian DNA replication, suggesting a direct involvement of H3K4me3, the main target of JARID1C, in early S-phase progression. Moreover, JARID1C silencing induces aberrant H3K4me3 levels at active origins, thus halting DNA replication and ultimately S phase progression. Together with the initiation defect observed upon JARID1C depletion, our data support an active role for H3K4me3 demethylation by JARID1C in DNA replication initiation. Our data also suggest that methylation of H3K4 is predominant on active, early-replicating origins. On late origins, instead, H3K4me1, 2 and 3 were undetectable. The analysis of the ENCODE dataset confirmed these results genome-wide, in HeLa and K562 cells. In all, these results suggest that the methylation of H3K4 exerts a major role specifically in the replication of early origins, and are in line with a recent study in yeast, that have shown a progressive loss of H3K4me3 during the S phase on replicating regions (32).

Defective DNA replication initiation may be due to defective licensing, as reported for knockdown of ORC, Cdc6, Ctd1 or MCM2–7 (63–65). Alternatively, it may result from unsuccessful origin firing, as reported for yeast initiation mutants (58,59). Interestingly, JARID1C knockdown hindered CDC45 loading on chromatin of S-phase cells. Our data are consistent with a recent report indicating a role of H3K4 methylation by MLL in preventing CDC45 binding to origins during checkpoint activation (74). Additionally, our results support the existence of a similar mechanism to regulate origin activation in the absence of checkpoint activation. In addition, we observed considerably less PCNA in association with chromatin. Importantly, demethylase defective JARID1C could not restore PCNA binding at early active origins nor abolish aberrant H3K4me3 from origins. These data suggest that JARID1C drives the efficient transition from pre-RC to origin firing through H3K4me3 demethylation and PCNA binding to activated origins.

Based on our data, silencing of JARID1C strictly phenocopies initiation mutants. Cells lacking JARID1C initiate DNA replication from fewer origins and have lower rate of BrdU incorporation. Of note, forks that manage to fire in absence of JARID1C do not undergo any elongation defect. All together, these evidence points to a direct role of JARID1C in the early events of DNA replication, namely initiation and origin firing. Data from yeast initiation mutants such as ORCs and Sic1 depleted strains indicate that impaired DNA replication initiation hinders S phase progression (58,59). In fact, the inability to activate some origins might significantly expand the distance a fork needs to cover.

In conclusion, here we show that JARID1C, an histone demethylase is required for proper DNA replication, by the removal of methyl groups from H3K4 residues on actively replicating early origins.

## SUPPLEMENTARY DATA

Supplementary Data are available at NAR Online.

SUPPLEMENTARY DATA
