# Supplementary material for: H3K4me3 demethylation by the histone demethylase KDM5C/JARID1C promotes DNA replication origin firing
Source: Nucleic Acids Res. 2015 Feb 23;43(5):2560–74. doi: 10.1093/nar/gkv090 (PMC4357704; doi:10.1093/nar/gkv090)
Supplement: SUPPLEMENTARY DATA [file supp_43_5_2560__index.html]

H3K4me3 demethylation by the histone demethylase KDM5C/JARID1C promotes DNA replication origin firing — H3K4me3 demethylation by the histone demethylase KDM5C/JARID1C promotes DNA replication origin firing — SUPPLEMENTARY DATA 

# H3K4me3 demethylation by the histone demethylase KDM5C/JARID1C promotes DNA replication origin firing

## SUPPLEMENTARY DATA

**Files in this Data Supplement:**

- Supplementary Figures
