# Supplementary material for: H3K4me3 demethylation by the histone demethylase KDM5C/JARID1C promotes DNA replication origin firing
Source: Nucleic Acids Res. 2015 Feb 23;43(5):2560–74. doi: 10.1093/nar/gkv090 (PMC4357704; doi:10.1093/nar/gkv090)
Supplement: SUPPLEMENTARY DATA [file supp_gkv090_nar-02346-m-2014-File007.pdf]

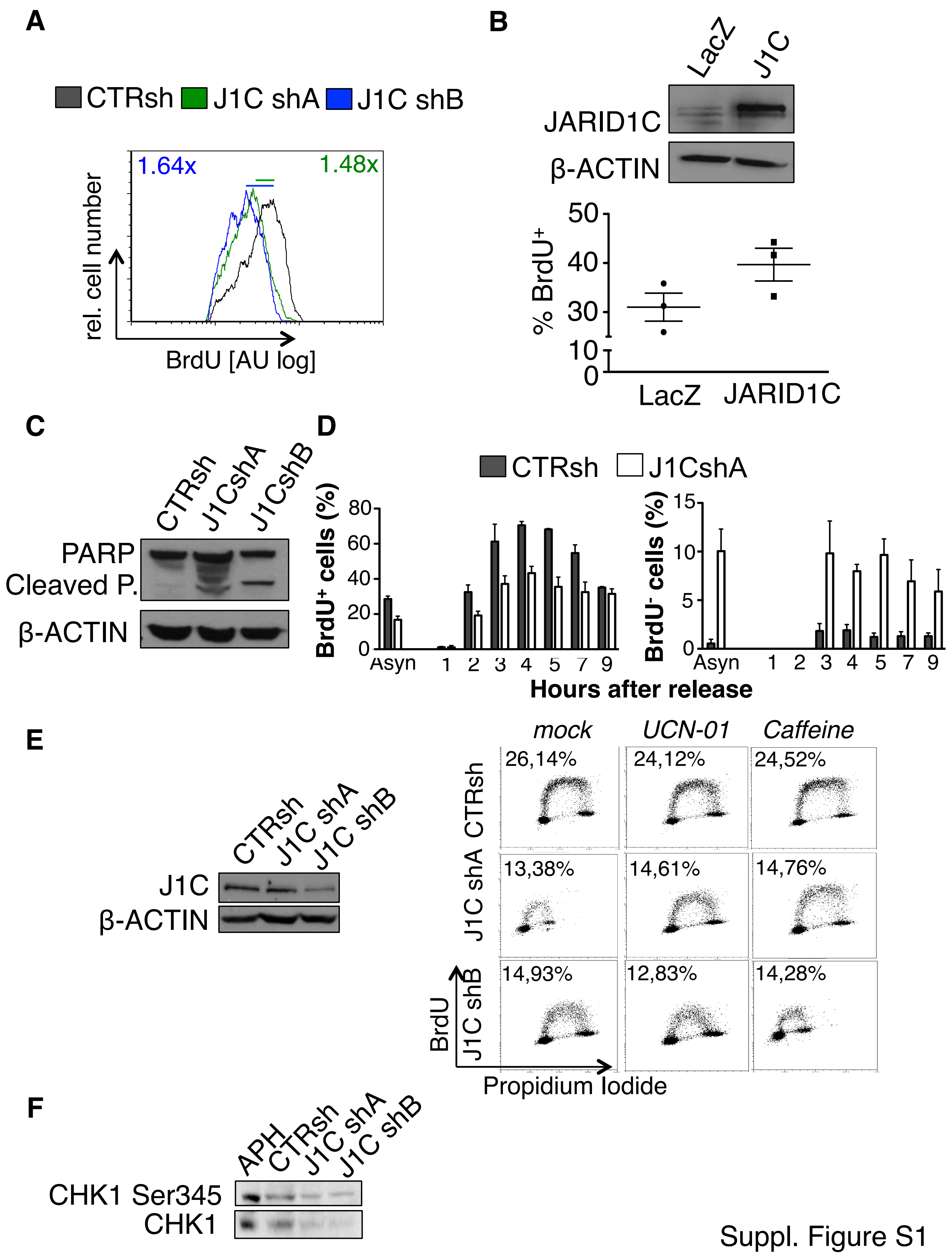

Suppl. Figure S1

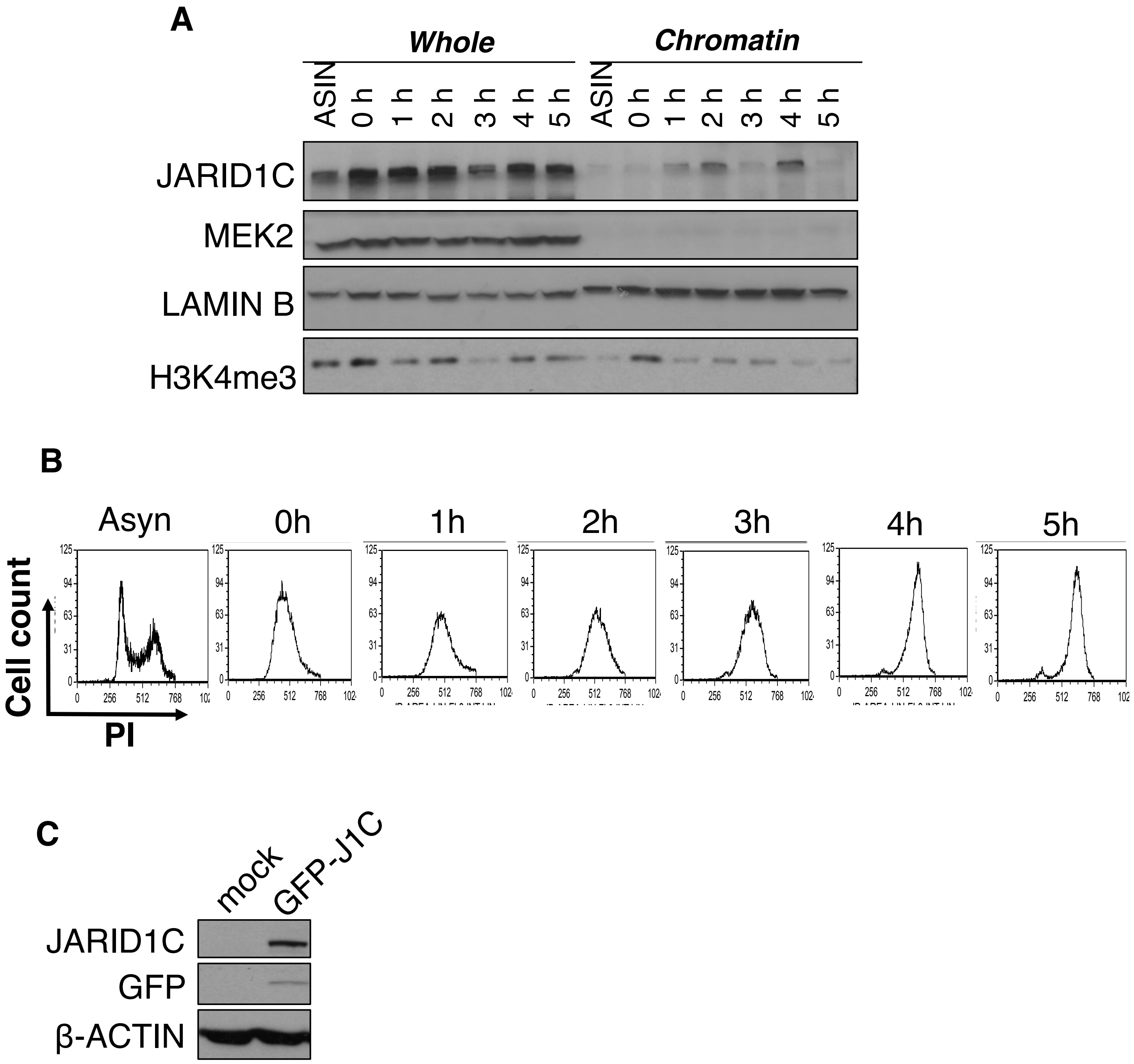

Suppl. Figure S2

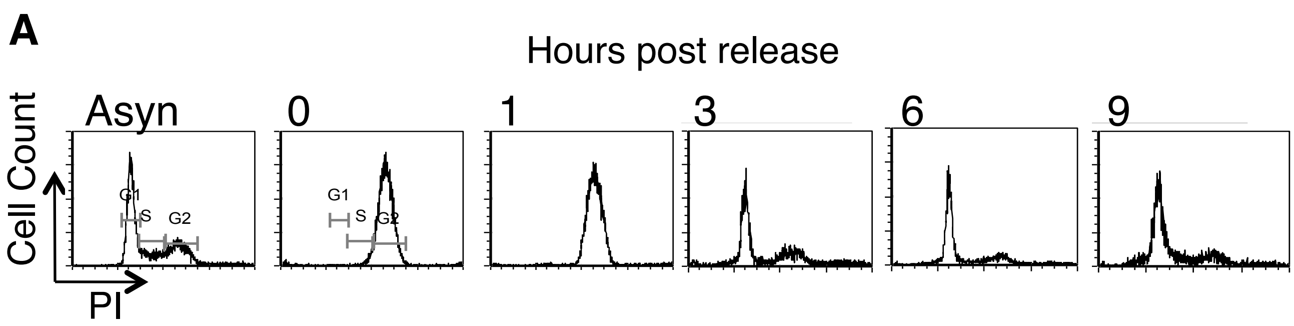

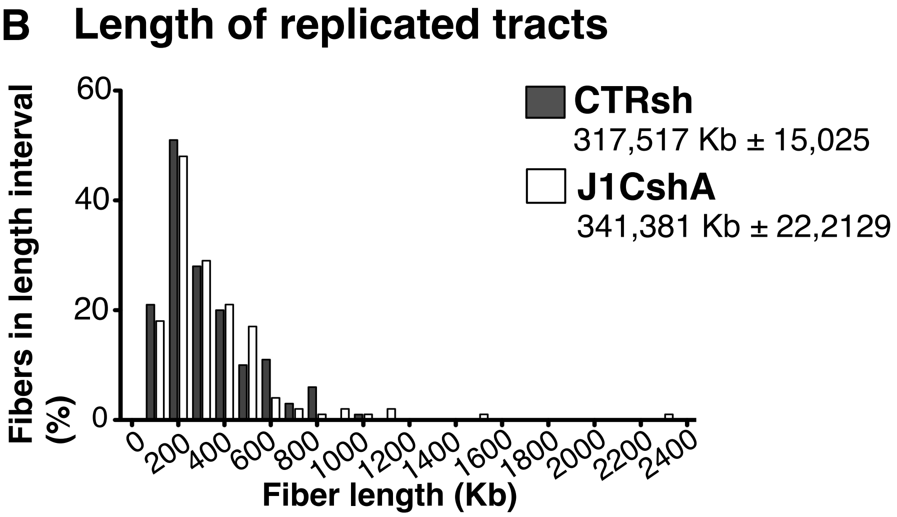

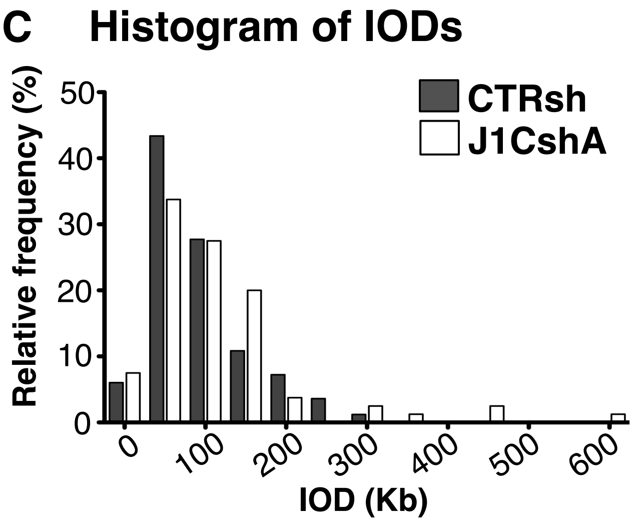

IOD (Kb)	CTRsh	J1CshA
n	83	80
Minimum	19	12
25% Percentile	47	56
Mean	92,1	112,4
75% Percentile	114	138
Maximum	281	592
Mean+/- Std.Error	92,1 +/- 6,624	112,4 +/- 11,218
Coeff. Variation (%)	65,5	89,3
Skewness	1,18	2,64

Suppl. Figure S3

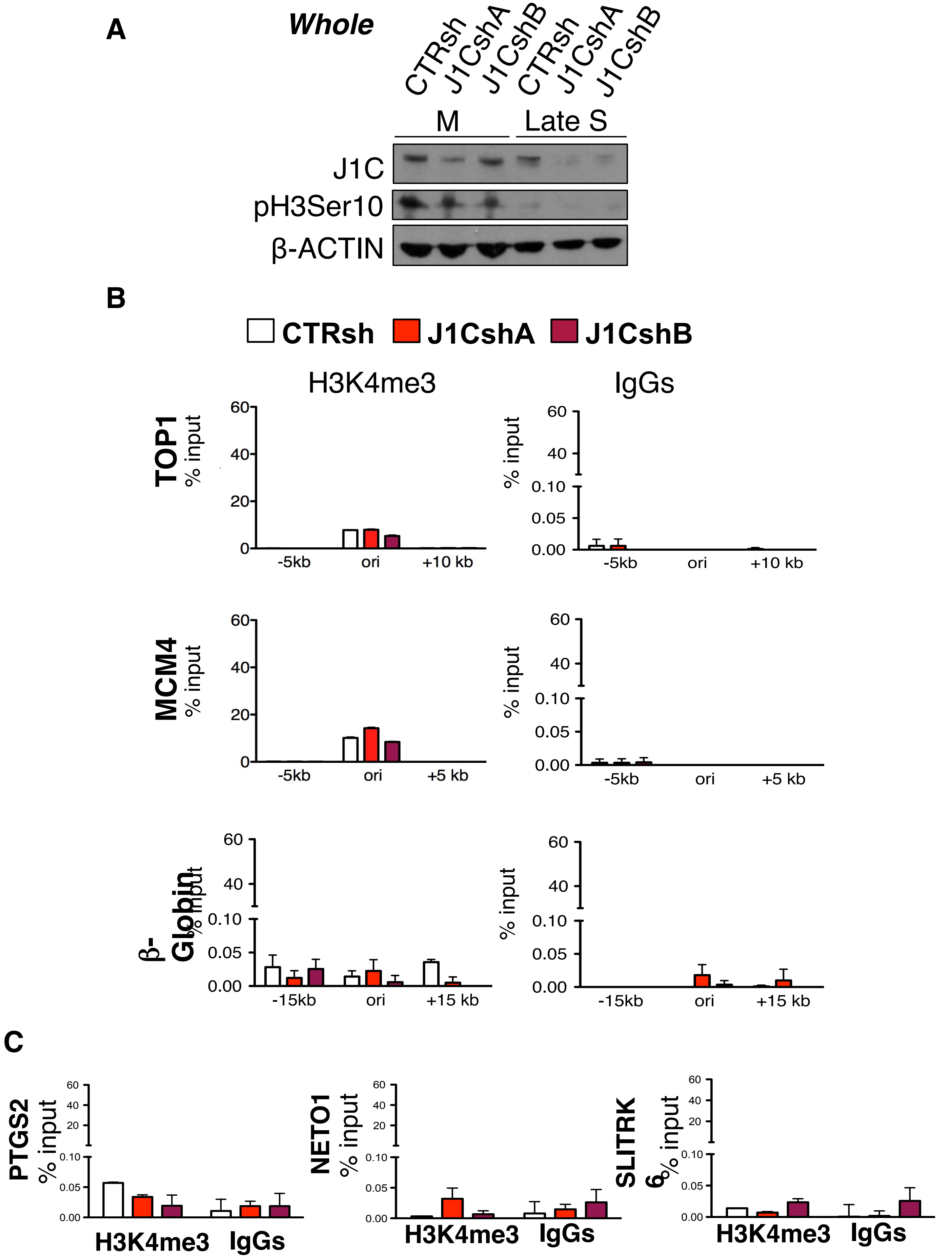

Suppl. Figure S4

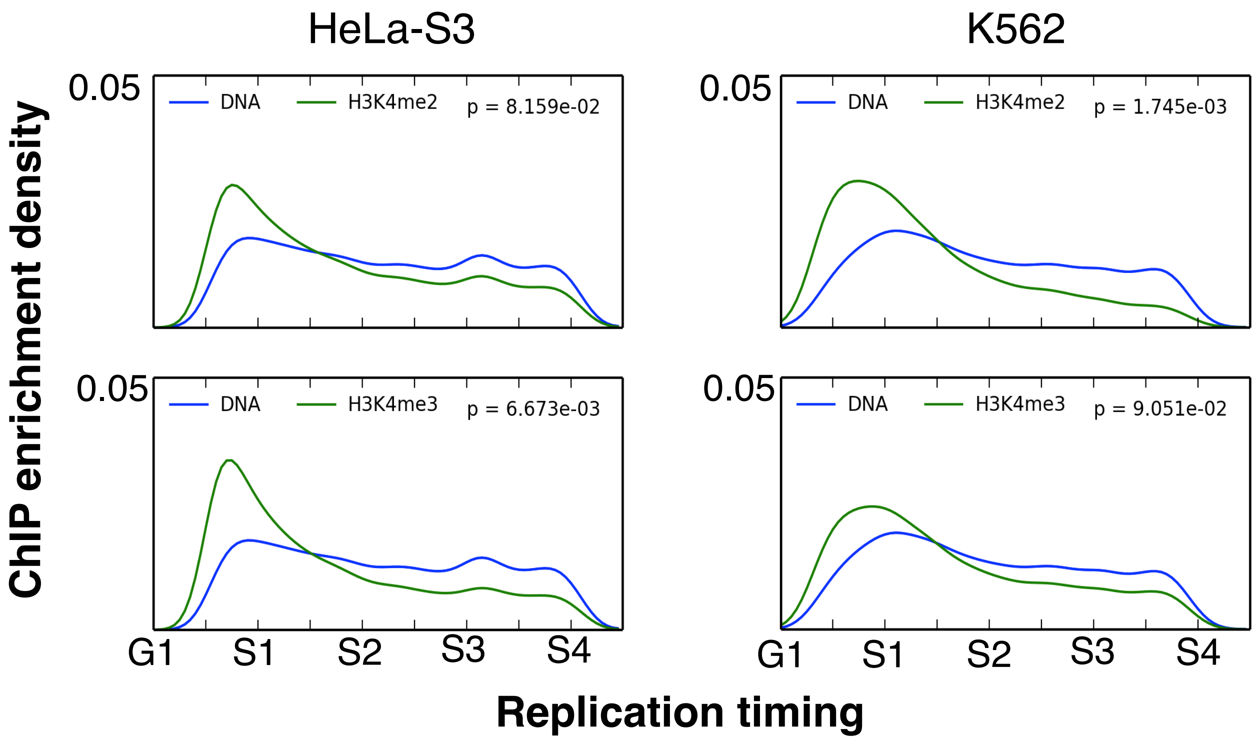

Suppl. Figure S5

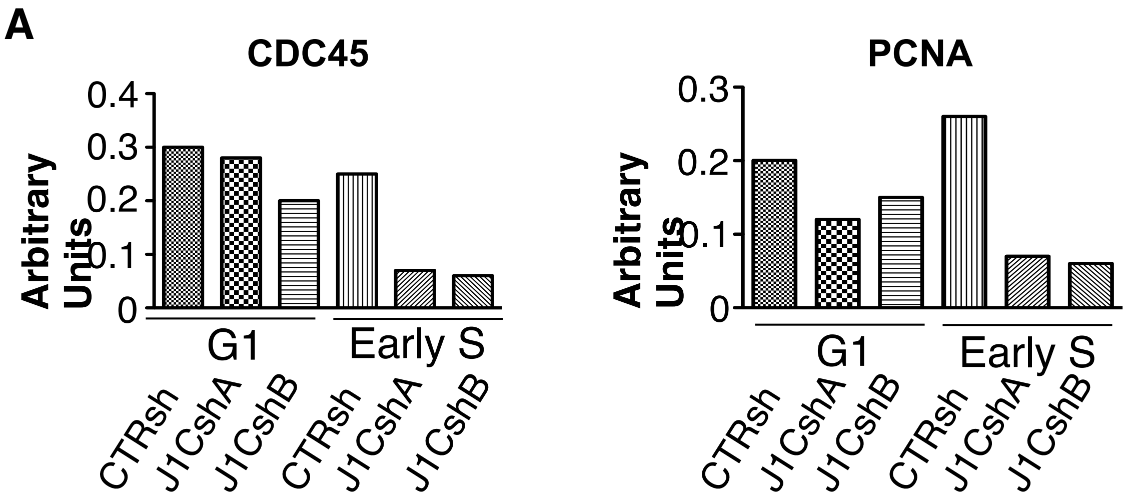
A
CDC45
PCNA
Arbitrary Units
0.4
0.3
0.2
0.1
0
G1
Early S
CTRsh
J1CshA
J1CshB

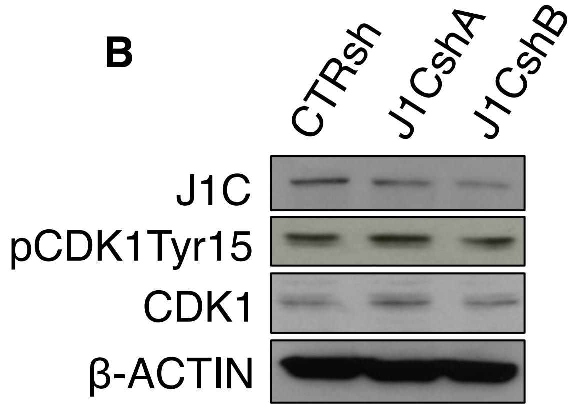
B
CTRsh
J1CshA
J1CshB
J1C
pCDK1Tyr15
CDK1
β-ACTIN

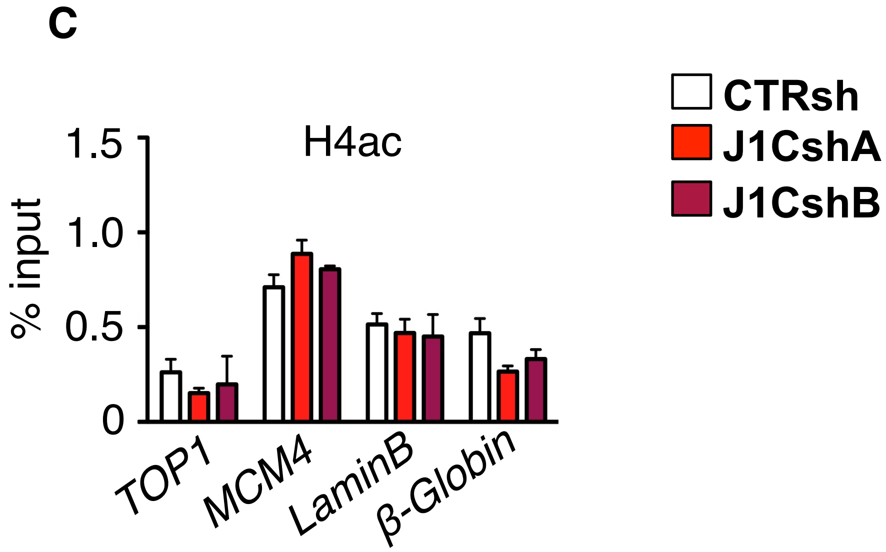
C
H4ac
% input
1.5
1.0
0.5
0
TOP1
MCM4
LaminB
β-Globin
CTRsh
J1CshA
J1CshB

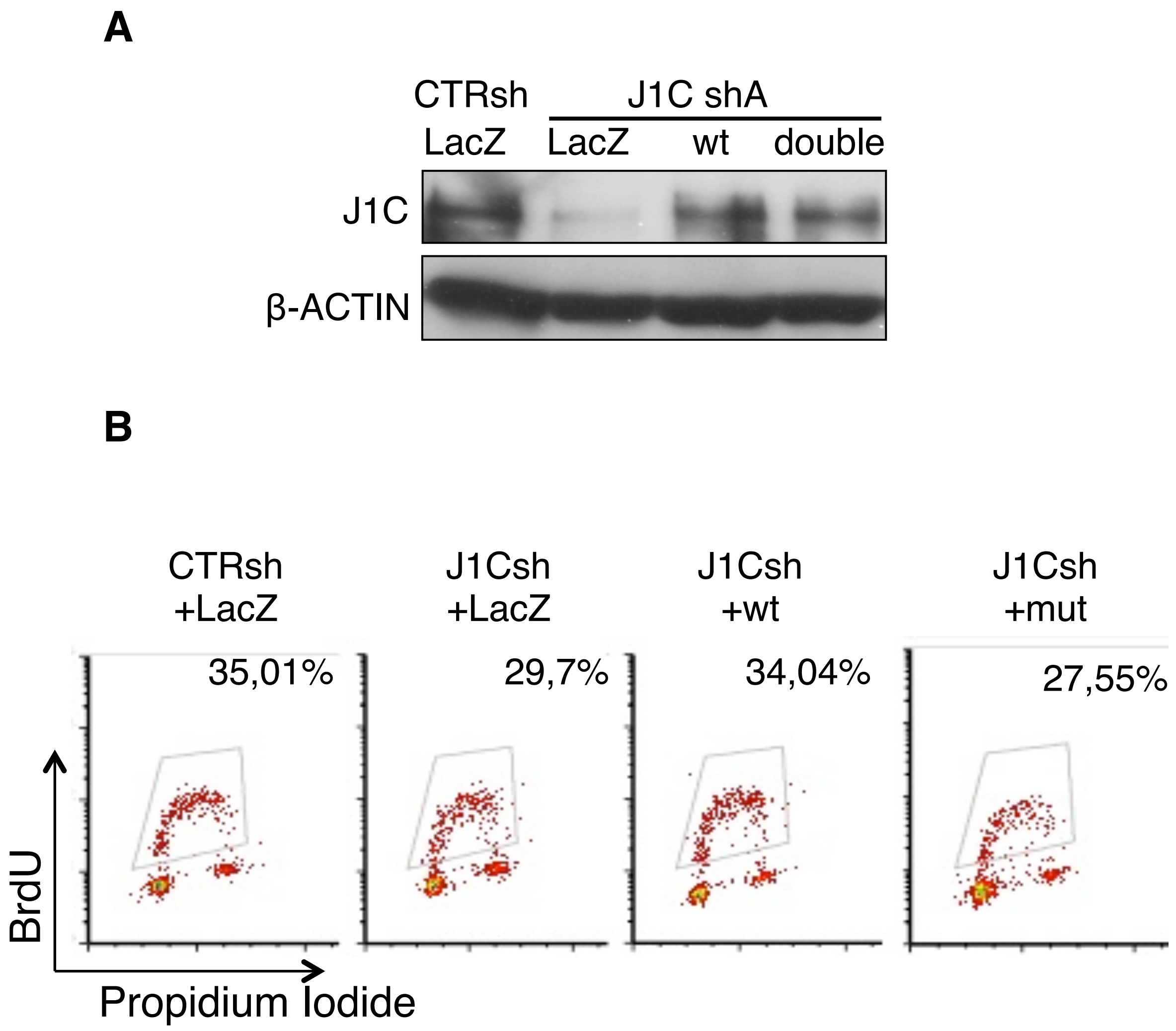

Suppl. Figure S7

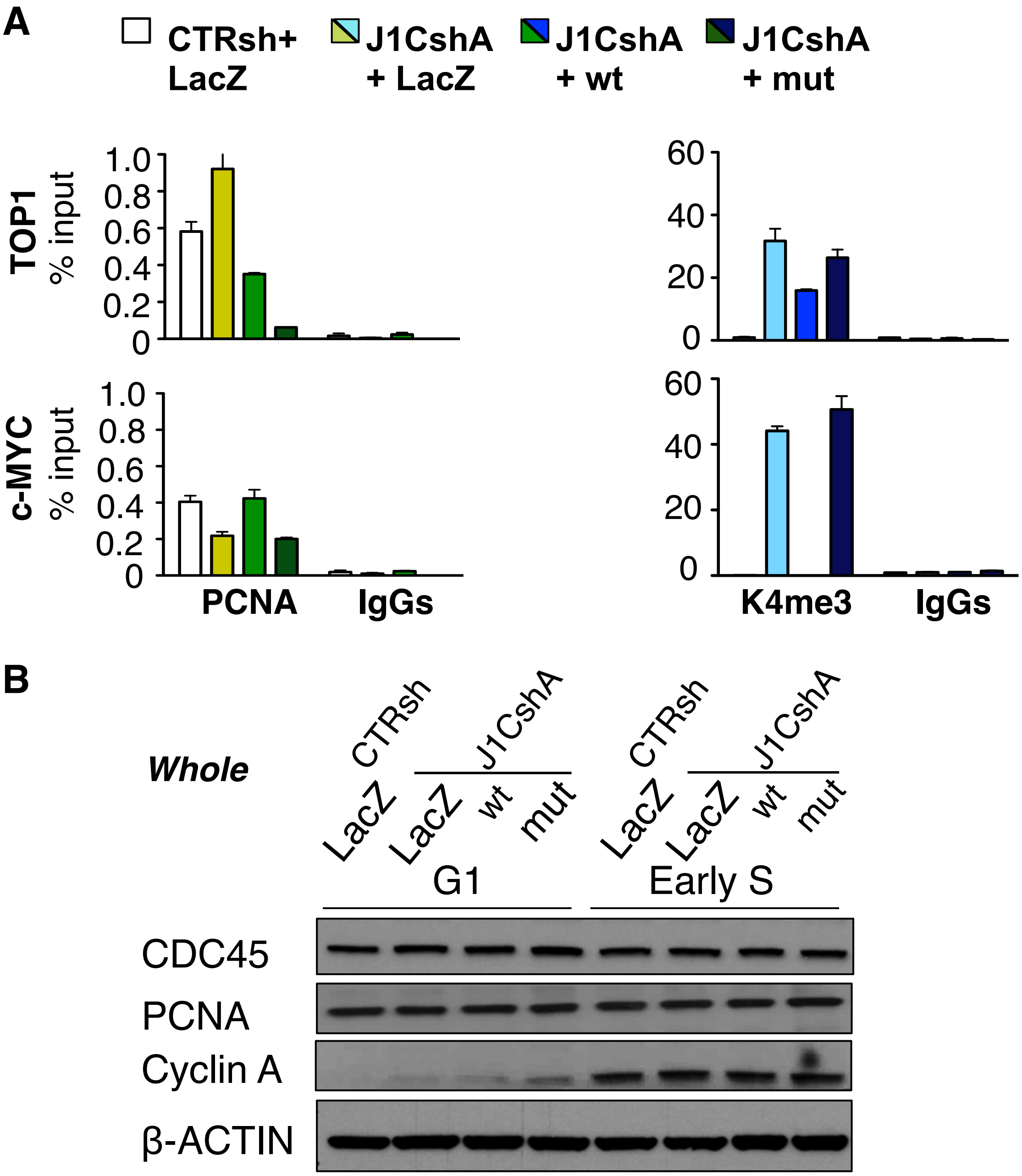
A
CTRsh+ LacZ
J1CshA + LacZ
J1CshA + wt
J1CshA + mut
TOP1
% input
1.0
0.8
0.6
0.4
0.2
0
60
40
20
0
c-MYC
% input
1.0
0.8
0.6
0.4
0.2
0
60
40
20
0
PCNA
IgGs
K4me3
IgGs
B
Whole
CTRsh
J1CshA
LacZ
LacZ
wt
mut
CTRsh
J1CshA
LacZ
LacZ
wt
mut
G1
Early S
CDC45
PCNA
Cyclin A
β-ACTIN

FIGURE LEGENDS

Supplementary Figure S1. (A) Histograms of S phase cells stained with an Alexa Fluor 647-coupled antibody to BrdU (AU, arbitrary units; log, logarithmic scale). 1.48x and 1.64x indicate fold decrease of BrdU fluorescence upon JARID1C knockdown for 72 hours as compared to control cells. **(B) Left**, expression of JARID1C and β-ACTIN in HeLa cells transfected with a LacZ control or a JARID1C expressing MSCV plasmid. **Right**, Quantification of BrdU incorporation upon mock LacZ or JARID1C transfection. **(C)** Western blot analysis of total cell lysate of control and J1C down-regulated cells. Both the full length and the cleaved form are detected with the anti-PARP1 antibody. β-ACTIN represents the loading control. **(D)** HeLa cells synchronized by double thymidine and released afterwards. Graphs depict percentages of control and BrdU positive (**left**) and negative (**right**) cells at different time points (hours) after release from thymidine block. Bars represent the average and SEM of two independent experiments performed. **(E) top,** Western blot analysis of JARID1C and β-ACTIN expression in whole cell lysates; **bottom**, BrdU incorporation of CTRsh, J1CshA and J1CshB cells mock treated or treated with caffeine or UCN-1 for 2 hours. A representative experiment of three is shown. **(F)** Western blot analysis of total cell lysate of control and J1C silenced cells. *APH* represents aphidicolin-treated cells, a positive control for CHK1 activation. The levels of both total CHK1 and CHK1 phosphorylated at Serine 345 are shown.

Supplementary Figure S2. (A) Western blot analysis of JARID1C and H3K4me3 in whole cell lysates and chromatin fractions of double thymidine G1/S synchronized HeLa cells. Chromatin bound fraction was obtained by CSK fractionation. MEK2 represents a cytosolic MAPK kinase and thus a fractionation control; lamin B represents the loading control. **(B)** Cell cycle distribution of HeLa cells after double thymidine block and release as shown in **(A)**. PI histograms are shown, with numbers depicting hours post release from the block. **(C)** Western blot analysis of whole cell lysates of HeLa cells mock transfected or transfected with GFP-JARID1C.

Supplementary Figure S3. CTRsh and J1CshA cells were sequentially labeled by IdU and CldU (thymidine analogues) and further analyzed by molecular DNA combing. **(A)** Cell cycle distribution of HeLa cells after nocodazole release. PI

histograms are shown, with numbers depicting hours post release from the block. **(B)** Lengths of replicated tracts are shown and occurrence events for the different classes were scored. **(C) left,** Histogram of CTRsh and J1CshA IOD measurements and relative frequencies are shown; **right,** Statistic of IOD measurements is reported. The number of measurements analyzed is n=83 and n=80 for CTRsh and J1CshA cells respectively. One representative DNA combing experiment is shown.

Supplementary Figure S4. (A) CTRsh and J1CshA/shB silenced cells were synchronized with nocodazole, released for 12 hours and harvested, corresponding to enrichment of cells in mitosis (M, 0 hours from release) and late S (12 hours from release). Total cell lysates were analyzed for expression of JARID1C, phosphorylated Serine 10 on histone 3 (pH3Ser10), and β-ACTIN. **(B)** Chromatin of late S control and down-regulated cells was immunoprecipitated with anti-H3K4me3 antibodies. Isotipic IgGs were used as controls. qPCR analysis on immunoprecipitated chromatin was performed with primers against *TOP1*, *MCM4* and *β-globin* DNA replication origins. Results are expressed as percentage of input (% input). The error bars represent SEM. **(C)** Analysis of H3K4me3 enrichment by ChIP as in **(B)** for three late-replicating human DNA replication origins, PTGS2, NETO1 and SLITRK6.

Supplementary Figure S5. Summarized coverage of H3K4me2 and me3 marks over replication timing in HeLa-S3 and K562 cells. Each panel represents the total amount of sequenced DNA obtained in ChIP-seq experiments for H3K4me marks binned by replication timing found by Repli-Seq. The distributions (green lines) are overlayed to the distribution of Input DNA for matched cell lines (blue lines). Regions associated to methylation of H3K4 are enriched in early S phase, compared to genomic DNA, and appear to be depleted in late S phase. P-values were calculated using one-way Kolmogorov-Smirnov test on two samples.

Supplementary Figure S6. (A) Quantification of CDC45 and PCNA chromatin bound levels reported in Figure 5A. See Materials and Methods for the experimental procedures. **(B)** Western blot analysis of JARID1C expression in total cell lysates of CTRsh, J1CshA and J1CshB cells. Total CDK1 and phosphorylation of inhibitory Tyrosine 15 levels (pCDK1Tyr15) are shown. β-ACTIN represents the loading control. **(C)** Chromatin of control (CTRsh) and silenced (J1CshA and J1CshB) cells

was immunoprecipitated with anti-H4 antibodies or isotipic IgGs. Enrichment of genomic regions corresponding to *TOP1*, *MCM4*, *Lamin B2* and *β-globin* replication origins was evaluated by qPCR. Results are expressed as percentage of input (% input). The error bars represent SEM.

Supplementary Figure S7. (A) JARID1C expression in total cell lysates of control and silenced cells was analyzed by Western blot upon rescue with LacZ, J1C wild type ("wt") or J1C mutant ("mut") rescue plasmids. Expression of β-ACTIN was used as loading control. **(B)** HeLa cells of complementation experiments in **(A)** were evaluated for cell cycle progression and BrdU incorporation by PI/BrdU fluorescence-activated cell sorting (FACS) as described in Materials and Methods.

Supplementary Figure S8. (A) Chromatin of rescued control and down-regulated cells was immunoprecipitated with anti-PCNA and anti-H3K4me3 antibodies. Isotipic IgGs were used as controls. qPCR analysis on immunoprecipitated chromatin was performed with primers against the *TOP1* and the *c-MYC* DNA replication origins. Results are expressed as percentage of input (% input). The error bars represent SEM of a representative experiment of two performed. **(B)** G1 and early S total cell lysates of complementation experiment shown in Figure 5 were analyzed for expression of the replication proteins CDC45 and PCNA and Cyclin A by Western blot. Expression of cyclin A was used to evaluate progression from G1 to S phase. Expression of β-ACTIN was used as a loading control.
